# Clinical applications of contactless photoplethysmography for vital signs monitoring in pediatrics: A systematic review and meta-analysis

**DOI:** 10.1017/cts.2023.557

**Published:** 2023-05-25

**Authors:** Melissa Bautista, Daniel Cave, Candice Downey, James R. Bentham, David Jayne

**Affiliations:** 1 University of Leeds, Leeds, West Yorkshire, UK; 2 General Surgery Department, St James’s University Hospital, Leeds Teaching Hospitals NHS Trust, Leeds, West Yorkshire, UK; 3 Leeds Children’s Hospital, Leeds Teaching Hospitals NHS Trust, Leeds, West Yorkshire, UK

**Keywords:** Vital signs monitoring, photoplethysmography, paediatrics, children, neonates, heart rate, respiratory rate, systematic review, meta-analysis

## Abstract

**Background:**

Contactless photoplethysmography (PPG) potentially affords the ability to obtain vital signs in pediatric populations without disturbing the child. Most validity studies have been conducted in laboratory settings or with healthy adult volunteers. This review aims to evaluate the current literature on contactless vital signs monitoring in pediatric populations and within a clinical setting.

**Methods:**

OVID, Webofscience, Cochrane library, and clinicaltrials.org were systematically searched by two authors for research studies which used contactless PPG to assess vital signs in children and within a clinical setting.

**Results:**

Fifteen studies were included with a total of 170 individuals. Ten studies were included in a meta-analysis for neonatal heart rate (HR), which demonstrated a pooled mean bias of −0.25 (95% limits of agreement (LOA), −1.83 to 1.32). Four studies assessed respiratory rate (RR) in neonates, and meta-analysis demonstrated a pooled mean bias of 0.65 (95% LOA, −3.08 to 4.37). All studies were small, and there were variations in the methods used and risk of bias.

**Conclusion:**

Contactless PPG is a promising tool for vital signs monitoring in children and accurately measures neonatal HR and RR. Further research is needed to assess children of different age groups, the effects of skin type variation, and the addition of other vital signs.

## Background

Photoplethysmography (PPG) is an optical technique which allows for measurement of parameters associated with cardiorespiratory function by detecting small blood volume changes within the skin [1]. Conventional PPG is widely used for pulse oximetry in most pediatric health care settings and requires contact with the skin. Camera-based PPG is an adaptation of this technique which uses a noncontact method of detecting these blood volume changes from a subject, with the use of a camera. This technique has gained interest due to the multiple advantages associated with noncontact methods of obtaining patient’s physiological parameters [2].

This technology is particularly interesting in pediatric populations due to the inherent advantages of covert vital signs measurements. It allows for less patient distress and eliminates the risk of Medical Adhesive Related Skin Injuries (MARSI) [3]. MARSI are common in neonatal populations due to their thin and delicate skin, with higher risk of iatrogenic skin damage [4].

Camera-based PPG has the potential to provide similar information to currently available contact-based methods. Heart rate (HR), respiratory rate (RR), and oxygen saturations are vital signs readily obtained with PPG and used frequently within health care settings as part of the ongoing assessments of patients [5,6]. With further understanding of the PPG waveform and its association with physiological parameters, other potentially useful parameters such as HR variability and perfusion index can also be extracted. These parameters have been correlated with other clinical markers and have been demonstrated to add diagnostic value; however, they are not currently used in every day clinical practice [7,8].

There is academic interest in application of camera-based PPG alongside refinement of algorithms to improve data acquisition. Multiple studies have been conducted using cameras to obtain PPG signals and vital signs. The majority have been conducted in a laboratory setting. Camera-based PPG use in real-life clinical settings is lacking [9]. Currently, there is minimal evidence on the accuracy and usability of camera-based PPG in pediatric populations including within clinical settings.

### Primary and Secondary Objectives

The primary objectives of this systematic review are to summarize current knowledge on the clinical application of camera-based PPG in pediatrics with the hope of stimulating discussion on future trial design to evaluate the usefulness of this methodology in different clinical pediatric settings.

For the purpose for this study, a clinical setting was defined as either a clinical environment where patient assessments occurred or the assessment of patients with a particular clinical condition even if this occurred in a laboratory setting. The secondary objectives are to assess the accuracy of camera-based PPG in different clinical settings and demonstrate any barriers to camera-based PPGs further evaluation in clinical environments.

## Methods

The search terms are available in supplementary material. This review adheres to the Preferred Reporting Items for Systematic reviews and Meta-analysis (PRISMA) guidelines.

### Inclusion and Exclusion Criteria

*Population*: The study was limited to pediatric patients under the age of 16 years. Given the broad range of ages within this population, the authors further divided the age groups into preterm neonates (born before 37 weeks gestation), neonates (0–28 days of age), infants (29 days – 1 year), and children (1–16 years).

*Intervention:* Contactless vital signs monitoring using camera-based PPG methods.

*Environments*: Only studies conducted within a clinical setting were included or pediatric patients with a particular medical condition and in any setting.

*Comparator:* The authors included all studies fitting the inclusion criteria with an available comparator from established contact methods of vital signs monitoring.

*Outcomes:* Outcomes included the detection rate and accuracy of any physiological parameters (HR, RR, saturation level, and blood pressure). Where available, further details regarding the acceptability, adverse events, and barriers were included.

*Exclusion*: Non-English studies, conference abstracts, protocols, and reviews were excluded.

### Search Strategy and Study Selection

An initial limited search was conducted to identify the appropriate search terms and available synonyms. The initial search terms were PPG, camera, and pediatrics. The final search terms can be found in appendix one.

Using a defined search strategy, OVID, WebOfScience, Cochrane library, and clinicaltrials.org were systematically searched by two independent investigators (MB and DC). The search was conducted from database inception to February 2022. All titles, abstracts, and then full texts were screened against the predetermined inclusion and exclusion criteria by the two investigators. The relevant research item abstracts were then reviewed to determine relevance. References of relevant review articles were also screened for other possible studies not picked up by the search terms. Comparing the final set of eligible papers between the two researchers (MB and DC) results in there being two discrepancies. These discrepancies were resolved through discussion.

### Data Extraction

Results from the independent literature searches were compared to form the final list of included studies. Data extraction was completed by two independent researchers (MB and DC) using a predetermined data extraction tool on Microsoft Excel (Microsoft Corporations, 2018). Extracted parameters included number of participants, participant demographics, vitals signs recorded, comparators, details of the technology used, and the results. Study findings included data on accuracy, signal quality, and other relevant observations such as any potential side effects.

### Risk of Bias Assessment

The authors used the Quality Assessment of Diagnostic Accuracy of Studies-2 (QUADAS-2) tool for risk of bias for comparative accuracy studies [10]. Two authors (MB and DC) independently completed the risk of bias assessment, and the results were compared, and discrepancies resolved through discussion. The risk of bias was completed for included studies and is shown in Supplementary Table 1.

### Data Analysis

The methods and results of included studies are described using a narrative approach. Additionally, a pooled statistical analysis was conducted where possible for studies with similar populations and outcome measures. Studies could be included in the pooled analysis if results were described using Bland–Altman analysis [11]. For relevant studies that did not report Bland–Altman analyses, values of mean bias and 95% limits of agreement (LOA) were derived from available data where possible. A random effects model was used to calculate pooled mean bias and 95% LOA, and these are displayed for each outcome using Forest plots (Stata 17.0 MP, StataCorp LLC, Texas, USA). Study weighting was preformed using the inverse variance method.

We considered a *p* value <0.1 as statistically significant for heterogeneity between studies. *I* ^2^ 25% was considered low heterogeneity, *I* ^2^ 50% as intermediate heterogeneity, and *I* ^2^ 75% as high heterogeneity.

## Results

### Search Results

**Figure 1.**
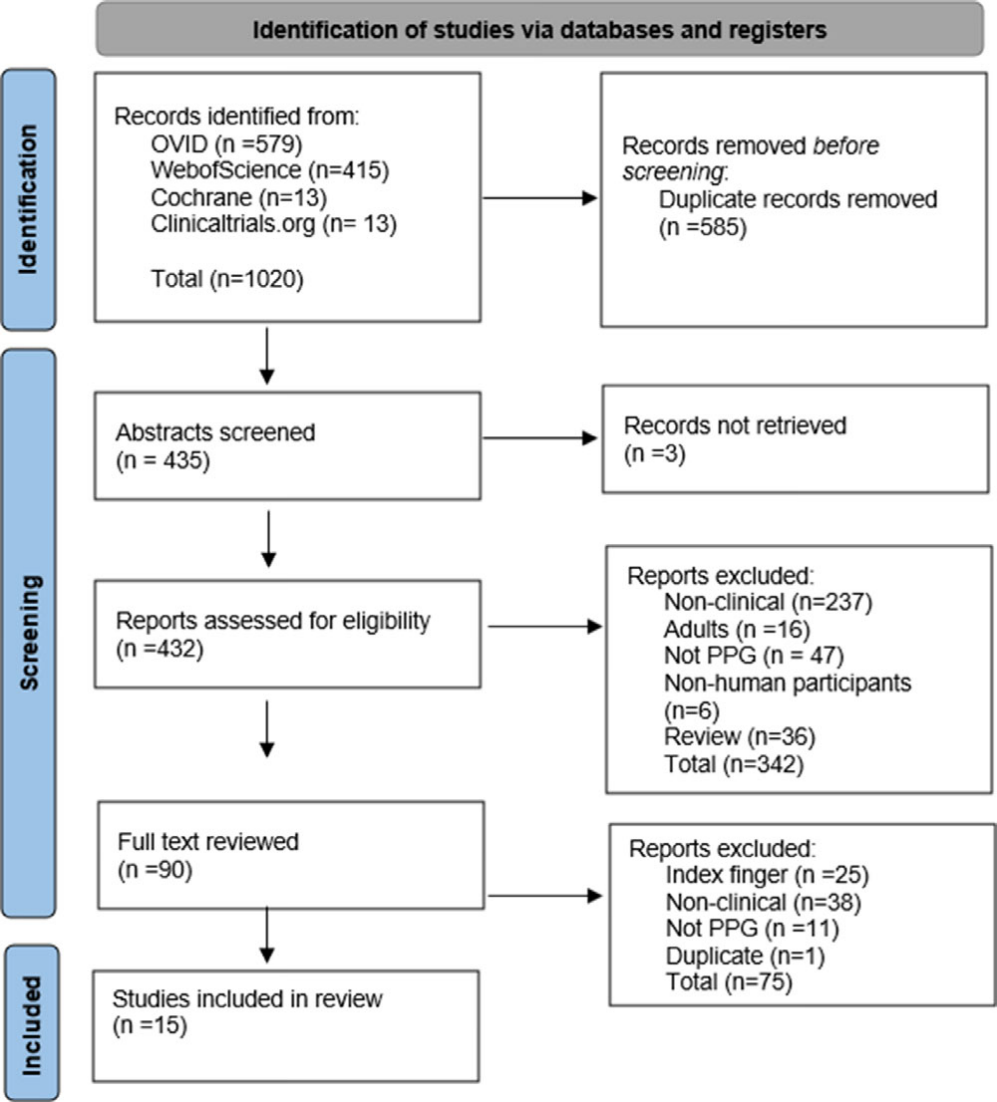
PRISMA flow diagram. PPG = photoplethysmography.

Of the four databases searched, a total of 435 abstracts were screened for eligibility. Following screening, 90 research articles with full text were reviewed and a total of 15 studies were then included in this review (Fig. 1). Forty-two percent of the studies were not considered clinical due to the use of healthy volunteers to either validate the technology or refine algorithms. Twenty-eight percent were also excluded due to the use of the index finger over a smart device camera, although this technology uses camera-based PPG, it is considered a contact method. Twelve percent of the studies used alternative methods of obtaining vital signs.

### Study Characteristics

A total of 15 studies and 170 individuals were included in the final review (Table 1). Most studies recruited neonatal intensive care inpatients (*n* = 14). Seven studies included a mixture of preterm and term neonates, three included preterm only, one included term only, and gestation was unknown in the remainder (*n* = 4). HR was the most frequently assessed vital sign (*n* = 12), followed by RR (*n* = 4) and oxygen saturation (*n* = 2). Eleven studies measured a single vital sign, and the remaining studies measured two. Studies varied in the imaging device used, with some using specialized red, green, and blue (RGB) cameras (*n* = 8) and others using digital cameras or web cameras (*n* = 7). The PPG algorithms used were different for each study.

**Table 1.** Description of included studies

Author, publication year, and country	Study size, *n*	Clinical setting	Population, gestation	Camera type (ROI)	Vital signs measured	Reference test
Scalise *et al*. (2012) [13] Italy	7	NICU	Neonates, 30–37 weeks	Digital camera (face)	HR	ECG
Aarts *et al*. (2013) [14] USA, The Netherlands	19	NICU	Neonates, 25–42 weeks	Digital camera (head, arm, thorax)	HR	ECG Pulse oximetry
Klaessens (2014) [15] The Netherlands.	7	NICU	Neonates – 87 days, 24–39 weeks	RGB camera (various ROI)	HR	ECG
Mestha *et al*. (2014) [16] India	8	NICU	Neonates, 37–40 weeks	Webcam (AI selected ROI - chest and face)	HR	Pulse oximetry
Bal *et al*. (2015) [5] Turkey	7	PICU	2 months – 14 years	Webcam (face)	HR, SO2	ECG Pulse oximetry
Cenci *et al*. (2015) [17] Italy	3	NICU	Neonates, 32–34 weeks	RGB camera (thorax, abdomen)	RR	ECG (impedance pneumography)
Van Gastel *et al*. (2016) [18] The Netherlands	2	NICU	Neonates	RGB camera (various ROI)	RR	ECG (impedance pneumography)
Blanik *et al*. (2016). [19]	10	NICU	Neonates (mean age 35 days), 24–28 weeks	RGB camera (head, hand, back)	HR	ECG Contact PPG
Jorge *et al*. (2017) [20] UK	30	NICU	Neonates, <37 weeks	Digital camera	Number of apnea events	ECG (impedance pneumography)
Antognoli *et al*. (2018) [6] Italy	7	NICU	Neonates, 26–40 weeks	Digital webcamera (thorax)	HR, RR	ECG
Cobos-torres *et al*. (2018) [21] Spain	9	NICU	Neonates, 25–40 weeks	Digital camera (diaphragm)	HR, RR	Unknown
Paul *et al*. (2020) [22] Germany	19	NICU	Neonates	Digital webcamera (various ROI)	HR	Pulse oximetry
Chen *et al*. (2020) [23] China	5	NICU	Neonates	RGB camera (face)	HR	ECG
Chen *et al*. (2021) [24] China	9	NICU	Neonates, 31–40 weeks	RGB camera (face)	HR	ECG
Wieler *et al*. (2021) [25] USA	28	NICU	Neonates, 36–41 weeks	Digital camera (forehead)	HR, SO2	Pulse oximetry

AI = artificial intelligence; ECG = electrocardiogram; HR = heart rate; LOA = limits of agreement; NICU = neonatal intensive care unit; PICU = pediatric intensive care unit; PPG = photoplethysmography; RGB = red green blue; ROI = region of interest; RR = respiratory rate; SO2 = oxygen saturation.

### ROI Selection

The selection of a region of interest (ROI) for PPG measurement demonstrated variation between the studies. Paul *et al.* used post-data acquisition analysis to find the ROI which provided the best quality signal, and this varied between participant and body part. Other studies used predetermined ROI with use of an automatic ROI detection method for obtaining PPG signals, with one study using artificial intelligence (AI) to detect the appropriate ROI [16]. This study used a varied number of ROIs which were governed by which ROI was available at different time points to account for loss of ROI due to motion. Overall, various ROIs were used across studies including the face, forehead, hands, arms, and thorax.

### Accuracy of Contactless PPG for Specific Clinical Applications

A summary of the results for included studies is shown in Table 2.

**Table 2.** Summary of findings table

Author, year	Study size, *n*	Vital signs measured	Reference test	Results (contactless PPG vs. reference)	Comments
Scalise *et al*. (2012) [13] Italy	*n* = 7	HR	ECG	Mean bias −0.9 bpm (LOA ± 8.9 bpm) Correlation coefficient 0.94	
Aarts *et al*. (2013) [14] USA, The Netherlands	*n* = 19	HR	ECG Pulse oximetry	Mean bias 0.3 bpm (LOA −5 to 5.6 bpm) Pulse oximetry vs. ECG: Mean bias −0.6 bpm (LOA −8.3 to 7.1 bpm)	Improved signal to noise ratio when used with phototherapy.
Klaessens (2014) [15] The Netherlands.	*n* = 7	HR	ECG	Good agreement with ECG (no values given)	Also measured RR by infrared thermography.
Mestha *et al*. (2014) [16] India	*n* = 8	HR	Pulse oximetry	Mean bias 2.5 bpm (LOA −3.0 to 8.0 bpm)	
Bal *et al*. (2015) [5] Turkey	*n* = 7	HR, SO2	ECG	HR: Mean bias −2.6 bpm (LOA −9.5 to 4.3 bpm) RMSE 4.1 Correlation 0.99 Accuracy 0.97 SO2: Correlation 0.71	Low hemoglobin resulted in underestimation of HR.
Cenci *et al*. (2015) [17] Italy	*n* = 3	RR	ECG (impedance pneumography)	Mean bias 1.0 (LOA −4.6 to 6.6)	
Van Gastel *et al*. (2016) [18] The Netherlands	*n* = 2	RR	ECG	Mean bias 0 (±20) MAE 4.7 RMSE 9.2 Correlation 0.89	
Blanik *et al*. (2016) [19]	*n* = 10	HR	ECG	Mean bias −2 bpm (LOA −21 to 17 bpm)	
Jorge *et al*. (2017) [20] UK	*n* = 30	Number of apnea events	ECG (impedance pneumography)	Detected 7 of 10 true apnea events	Reduced false alarm rate of 77%
Antognoli *et al*. (2018) [6] Italy	*n* = 7	HR, RR	ECG	HR: Mean bias −1.0 bpm (−10.8 to 8.8 bpm) RMSE 12.2 RR: Mean bias 0.3 (−5.8 to 6.4) RMSE 7.6	
Cobos-torres *et al*. (2018) [21] Spain	*n* = 9	HR, RR	Unknown	HR: Mean bias −1.5 bpm (−9.7 to 6.8 bpm) Correlation 0.94 RR: Mean bias 0.6 (−9.2 to 10.3) Correlation 0.86	
Paul *et al*. (2020) [22] Germany	*n* = 19	HR	Pulse oximetry	Mean bias −0.2 bpm (−2.3 to 1.9 bpm) RMSE 1.1	
Chen *et al*. (2020) [23] China	*n* = 5	HR	ECG	MAE 7.4 bpm RMSE 15.2	
Chen *et al*. (2021) [24] China	*n* = 9	HR	ECG	Mean bias −0.8 (−5.5 to 3.9) MAE 3.4 bpm	
Wieler *et al*. (2021) [25] USA	*n* = 28	HR, SO2	Pulse oximetry	HR: Mean bias −4.2 bpm (LOA −48.0 to 39.6 bpm) MAE 15.6 bpm RMSE 20.4 SO2: 75% sensitivity for detecting desaturation	

ECG = electrocardiogram; HR = heart rate; LOA = limits of agreement; MAE = mean absolute error; PPG = photoplethysmography; RMSE = root mean squared error; RR = respiratory rate; SO2 = oxygen saturation.

#### Neonatal HR measurement

Neonatal HR measurement was measured in the clinical environment under visible light for all studies. Some studies measured HR continuously over a prolonged period [16], while others acquired data intermittently, over a set period of time [13,14]. All studies used post-data acquisition processing to obtain the measured vital signs. Electrocardiogram (ECG) monitoring was used as the gold standard reference test in 8 out of 12 (67%) studies of HR measurement.

Aarts *et al.* used a digital camera to measure HR in 19 neonates under ambient light [14]. Videos of 1–5 minutes were taken, and then HR analyzed from various manually selected ROIs including the head, arm, and thorax. Authors demonstrated excellent agreement between contactless PPG and simultaneous ECG monitoring, with mean bias of 0.3 bpm and 95% LOA −5.0 to 5.5. Interestingly, contactless PPG was superior to pulse oximetry for HR measurement when both were compared to ECG [14].

**Figure 2.**
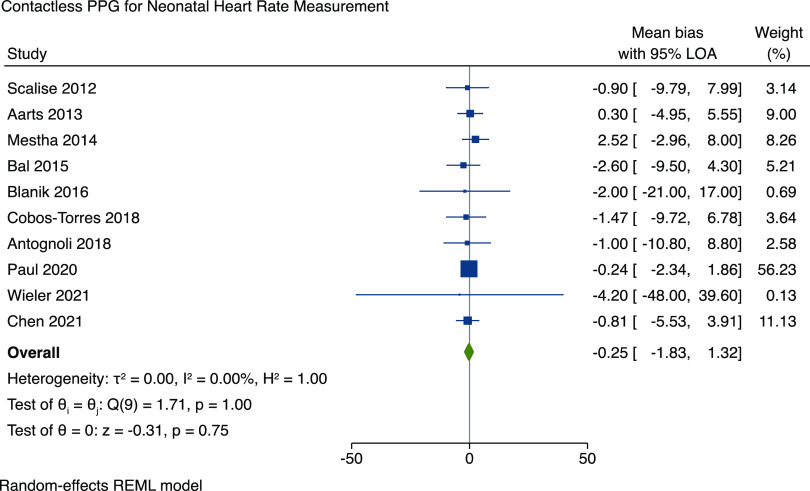
Forest plot demonstrating the accuracy of contactless PPG monitoring for heart rate. PPG = photoplethysmography; LOA = limits of agreement; REML = restricted maximum likelihood.

We performed a meta-analysis to assess the accuracy of contactless PPG for HR measurement across all studies. The mean bias and 95% LOA were pooled across studies and were manually calculated from data provided, where possible. Two studies did not report the raw data, mean bias, or Bland–Altman plots and therefore could not be included in the meta-analysis. The results are shown in Fig. 2 and demonstrate a pooled mean bias of −0.25 (95% LOA −1.83 to 1.32) with an *I* ^2^ value for heterogeneity of 0% (95% confidence interval 0 to 62%). A sensitivity analysis was conducted with Paul *et al*. (2020) removed, and the significance of the results remain unchanged, with a pooled mean bias of −0.27 (95% LOA of −2.65 to 2.11) (Figure 1 in supplementary material).

#### Neonatal RR measurement

**Figure 3.**
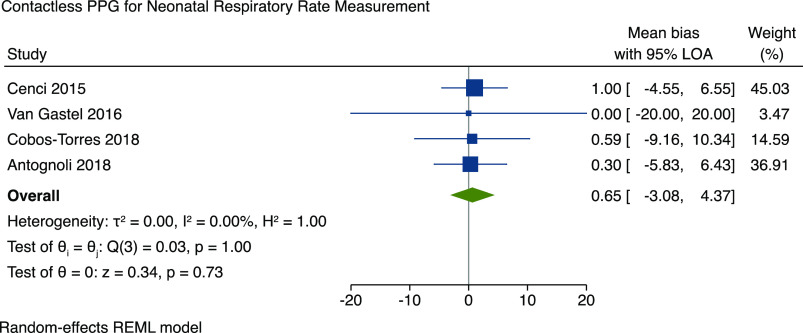
Accuracy of contactless PPG for respiratory rate. PPG = photoplethysmography; LOA = limits of agreement; REML = restricted maximum likelihood.

Four studies included methods for RR measurement in neonates using contactless PPG. Participants included preterm and term neonates that were studied during NICU admissions. One further study by Jorge *et al*. investigated the use of contactless PPG for the detection of apneic events in 30 preterm neonates [20]. Authors used a digital camera positioned over the incubator and were able to correctly detect 7 out of 10 apneic events and 34 of 44 artefactual events that would have triggered a false alarm with conventional monitoring. The majority of studies used impedance pneumography (IP) from ECG leads as the reference gold standard. All four studies reported the mean bias of RR measurement and could be included in the meta-analysis (Fig. 3). The results demonstrate a pooled mean bias of 0.65 (95% LOA of −3.08 to 4.37) and *I* ^2^ of 0% (95% confidence interval 0 to 85%).

#### Vital signs monitoring in children

One study recruited older children during pediatric intensive care (PICU) admissions. Bal *et al.* studied seven children aged 2 months to 14 years, and contactless PPG measurement of HR and oxygen saturations (SO2) was performed with a digital webcam [5]. Automated face detection followed by skin detection was used to identify the ROI, and PPG signal extracted from RGB pixel intensity. The mean bias for HR measurement was 2.6 bpm with LOA of ±6.9 bpm [5]. SO2 was estimated from red and blue signals using a “ratio of ratios” approach, and the authors demonstrated a strong correlation (correlation coefficient 0.71) in PICU patients.

### Effects of Ethnicity and Skin Color

Wieler *et al.* reported Fitzpatrick skin color scales for the participants in their study, with one participant being V-VI. They conclude that there were too few skin type variations to allow for meaningful comparisons [25]. Paul *et al.* acknowledged the skin color variations between North and South Indian participants but did not provide a subgroup analysis of the potential impact of skin color variation [22]. Aarts *et al.* reported one participant with darker skin had a reduced signal-to-noise ratio (SNR); however, this did not compromise the ability to pick up HR [14]. No other studies reported skin color or ethnicity of included participants.

### Risk of Bias

Following application of the QUADAS-2 tool to the studies, all studies were fully paired, but the majority lacked methodological and selection details to ensure accurate risk assessment and were assigned a moderate risk of bias. A common theme for the research studies included in the review was the selection of the best quality signal or results prior to comparison. A summary of the risk of bias assessment for each individual study is included in Fig. 2 of the supplementary material. Publication bias assessment was conducted by visualization of the funnel plot, which included studies measuring neonatal HR (Figure 3 supplementary material). On review of the funnel plot, there appears to be no publication bias. This was further supported by Egger’s test (*p* = 0.476) and Begg’s test (*p* = 0.531).

## Discussion

This systematic review summarizes the current evidence for contactless PPG vital signs monitoring in pediatric patients. Using camera-based contactless monitoring in pediatric populations may be an accurate and safe alternative for measuring HR in children, especially neonates. This review demonstrated good accuracy between the contactless PPG methods compared to the current gold standards for both HR and RR monitoring in neonates. Contactless PPG may even be superior to conventional pulse oximetry for HR measurement [14]. There were no studies using contactless PPG to measure blood pressure or alternative physiological parameters such as HR variability, methods known to be achievable with contactless PPG in adults [26].

There were no reports of adverse events related to the use of contactless PPG monitoring. Thermoregulation is an important factor, particularly in neonatal monitoring. Many of the included studies proposed a system for monitoring neonates in the incubator, with the camera set up externally, and reported good image quality and accuracy [11]. Any form of contactless PPG monitoring does require adequate ambient light and therefore may not be suitable for the continuous monitoring of preterm neonates in dark conditions. Infrared monitoring may offer an alternative solution for contactless monitoring in this population [12]. Another important feature is the ability of contactless PPG to detect clinical deterioration. Jorge *et al*. studied the detection of apneic events and proposed an algorithm that reduced the false alarm rate by 77% [20]. Future studies are required to ensure that contactless PPG is accurate over the entire range of neonatal HRs.

For clinical application, camera-based PPG methods must be accurate for all ethnicities and skin types. Conventional pulse oximeters using contact PPG can systematically overestimate the SO2 in patients of Black and Asian ethnicity, leading to delays in the detection of hypoxemia and escalation in management [27,28]. In our review, studies had small sample sizes and could not draw conclusions on the effects of skin-type variation and ethnicity on the accuracy of this technology. Wieler *et al.* reported the Fitzpatrick skin-type scale of neonates undergoing HR measurement; however, only one participant had skin-type V-VI [25]. Aarts *et al.* studied one neonate of Hispanic ethnicity (Fitzpatrick type V) and were successfully able to measure HR despite a relatively lower signal strength [14]. Two studies of infants with darker skin types reported excellent agreement between contactless PPG and pulse oximetry [14,16]. There is a need for further study of the effects of skin type on contactless PPG signal strength and accuracy.

Various other factors can influence the signal strength and measurement accuracy of contactless PPG. The brightness of ambient light is a commonly reported factor, and low light can result in reduced SNR [14]. One study reported an infant under bright phototherapy light had the highest signal quality, resulting in perfect correlation of HR measurement with ECG monitoring, however the authors fail to provide a supporting value [14], making the interpretation of this result difficult. Many studies also reported the challenges of patient movement and the ability to detect the measured vital sign [14,19]. The difficulty lies in the inability to always keep ROI in the frame, and this motion artifact reduces SNR and therefore quality of the PPG waveform. One study observed an underestimation in HR measurement for children with lower hemoglobin levels [5]. Improvements in image resolution and algorithm developments are likely to reduce the influence of environmental factors.

Contactless vital signs monitoring has many advantages for use in clinical practice, including the avoidance of skin probes that may cause skin damage in neonates. The technology has significant potential for clinical application in infants and children, avoiding distress during measurement that may cause falsely abnormal vital signs. Preliminary clinical studies have reported good accuracy for neonatal HR and RR measurement, and this is demonstrated in our meta-analyses (Figs. 1 and 2). There remain issues with movement artifact, and there is a lack of standardization between available devices. With further development, this technology may have a wide variety of clinical applications in pediatric inpatient and outpatient settings.

### Limitations

Included studies were mostly small, with variation in the camera used and image processing methods. On individual review of studies, most had a risk of bias and this was often related to unclear image processing methods or manual ROI selection, with no mention of blinding of the assessors. There was variation in the reporting of results and statistical methods, and therefore we were not able to include all studies in the meta-analyses. Those studies included in the meta-analyses used mean bias as the statistical method. This method of assessing differences has limitations, including an underestimation of difference where positive and negative values cancel out. This method was chosen as it was the most common statistical method provided by the included studies and therefore gave the largest amount of data to perform a meta-analysis. Most studies used ECG monitoring as the gold standard for HR monitoring; however, some used pulse oximetry which may also be affected by movement or skin type. Four out of the 15 studies reported funding sources (Table 2; Supplementary material), making it difficult to assess for funding bias; however, only one study claimed a potential conflict of interest. Future studies should prioritize automated methods of ROI selection and ensure the most suitable reference test is used. To aid clinical translation, technologies should prioritize real-time data acquisition for use in patient monitoring.

### Conclusion

Contactless PPG is a promising tool for vital signs monitoring in children and accurately measures neonatal HR and RR without the need for a skin probe. However, a lack of standardization in the algorithms and reporting of results means comparison between studies is challenging. Further research is needed to assess children of different age groups, the effects of skin-type variation, and the addition of other vital signs.

## Supplementary material

For supplementary material accompanying this paper visit https://doi.org/10.1017/cts.2023.557. click here to view supplementary material
