# Supplementary material for: Clinical applications of contactless photoplethysmography for vital signs monitoring in pediatrics: A systematic review and meta-analysis
Source: J Clin Transl Sci. 2023 May 25;7(1):e144. doi: 10.1017/cts.2023.557 (PMC10310860; doi:10.1017/cts.2023.557)
Supplement: Supplementary file 1 [file S2059866123005575sup001.docx]

Supplementary material

Search terms:

(OR) Photoplethysmography OR PPG OR iPPG OR rPPG
AND

Vital signs OR heart rate OR HR OR monitoring OR Oxygen Levels OR Oxygen Saturations OR Blood pressure OR BP OR Respiratory rate OR respiration OR RR

AND

Contactless OR Camera-based OR camera

**Supplementary Figure 1**. Sensitivity analysis of PPG Neonatal heart rate measurement (Paul et al 2020 removed)

*PPG, Photoplethysmography; LOA, limits of agreement; REML, restricted maximum likelihood*

**Supplementary Figure 2.** QUADAS-2 risk of bias assessment diagram.

**Supplementary Figure 3.** Funnel plot of studies measuring neonatal heart rate

*PPG, photoplethysmography*

**Supplementary Table 1** Risk of bias assessment (QUADAS-2 tool)[24].

| **Study** | **Risk of bias**  **(QUADAS-2)** | | | |
| --- | --- | --- | --- | --- |
|  | **P** | **I** | **R** | **FT** |
|  |  |  |  |  |
| Scalise, 2012 | ? | ✗ | ✓ | ? |
| Aarts, 2013 | ✓ | ? | ✓ | ✓ |
| Mestha, 2014 | ✓ | ? | ? | ✓ |
| Klaessens, 2014 | ✓ | ✗ | ✓ | ✓ |
| Bal, 2015 | ✓ | ? | ✓ | ? |
| Cenci, 2015 | ✓ | ✗ | ? | ✓ |
| Blanik, 2016 | ? | ✗ | ✓ | ? |
| Van Gastel, 2016 | ✓ | ? | ✓ | ✓ |
| Jorge, 2017 | ✓ | ? | ✓ | ✓ |
| Cobos-torres, 2018 | ? | ✗ | ? | ? |
| Antognoli, 2018 | ? | ✓ | ? | ✓ |
| Paul, 2020 | ? | ✗ | ? | ✗ |
| Chen, 2020 | ✓ | ? | ✓ | ✓ |
| Weiler, 2021 | ✓ | ✓ | ? | ? |
| Chen, 2021 | ✓ | ✓ | ✓ | ✓ |

P = patient selection; I = index test; R = reference standard; FT = flow and timing.
✓ indicates low risk; ✗ indicates high risk; ? indicates unclear risk.

**Supplementary Table 2.** Review of funding sources for each study

| **Author, publication year, country** | ***Funding source*** |
| --- | --- |
| Scalise et al. (2012).[11]  Italy | Not reported |
| Aarts et al. (2013).[12]  USA, The Netherlands | Not reported |
| Klaessens (2014).[13]  The Netherlands. | Not reported |
| Mestha et al. (2014).[14]  India | Not reported |
| Bal et al. (2015).[5]  Turkey | Not reported |
| Cenci et al. (2015).[15]  Italy | Not reported |
| Van Gastel et al. (2016).[16]  The Netherlands | IMPULS-II Programe for Data science flagship project |
| Blanik et al. (2016).[17] | Not reported |
| Jorge et al. (2017).[18]  UK | Wellcome Trust and EPSRC |
| Antognoli et al. (2018).[6]  Italy | Not reported |
| Cobos-torres et al. (2018).[19]  Spain | No external funding |
| Paul et al. (2020).[20]  Germany | BMBF and ICMR |
| Chen et al. (2020).[21]  China | Not reported |
| Chen et al. (2021).[22]  China | Shanghai Municipal Science and Technology Major Project and Partly by Philips |
| Wieler et al. (2021).[23]  USA | Not reported |

*EPSRC, Engineering and Physical Sciences Research Council; BMBF,* Germany’s Federal Ministry of Education and Research*;* ICMR, Indian Council of Medical Research
